# Protective Effects of Total Alkaloids of Rhizoma Corydalis Against Neuronal Ferroptosis Through a PPARγ-Dependent Mechanism in Diabetes-Associated Cognitive Impairment

**DOI:** 10.3390/ph19101506

**Published:** 2026-09-22

**Authors:** Yazhi Qi, Huasen Yang, Zhoujing Shi, Xingtong Chen, Mingshuang Sun, Pinghan Qi, Yufan Lin, Yusheng Han, Boyan Ma

**Affiliations:** Basic Medical College, Heilongjiang University of Chinese Medicine, Harbin 150000, China

**Keywords:** total alkaloids of rhizoma corydalis, PPARγ, cognitive dysfunction, diabetes, ferroptosis

## Abstract

**Objectives:** Previous studies from our laboratory have established a strong correlation between diabetes-associated cognitive impairment (DACI) and neuronal ferroptosis. This study aimed to elucidate the therapeutic mechanisms of total alkaloids of rhizoma corydalis (TAC) in mitigating DACI, with a specific focus on its modulation of neuronal ferroptosis via the PPARγ pathway. **Methods:** Therapeutic targets were predicted utilizing bioinformatics, molecular docking, and single-cell RNA sequencing (scRNA-seq) analyses. In vivo, a DACI rat model was established via a high-fat/high-sugar diet coupled with streptozotocin (STZ) intraperitoneal injection, followed by TAC administration. In vitro, HT22 cell injury was induced by palmitic acid and high glucose (PA + HG) exposure. Small interfering RNA (siRNA)-mediated PPARγ knockdown was employed to validate the protective mechanisms of TAC. **Results:** Multi-omics and computational analyses identified neuronal PPARγ as the central target mediating the anti-ferroptotic effects of TAC. In vivo assessments demonstrated that TAC administration significantly attenuated spatial memory deficits, dyslipidemia, and insulin resistance in diabetic rats. Furthermore, TAC mitigated hippocampal neuronal injury and mitochondrial morphological abnormalities, decreased iron overload and lipid peroxidation, and normalized the expression profiles of ferroptosis-associated proteins. In vitro assays, corroborated by siRNA-mediated knockdown, confirmed that TAC specifically upregulates PPARγ, which subsequently activates the Nrf2/HO-1 signaling axis and enhances GPX4 expression, ultimately conferring resistance against neuronal ferroptosis. **Conclusions:** TAC emerges as a highly promising therapeutic candidate for DACI, exerting its neuroprotective efficacy intrinsically through the targeted activation of PPARγ to abrogate neuronal ferroptosis.

## 1. Introduction

Diabetes mellitus has reached pandemic proportions globally and is intricately linked to central nervous system complications [1,2]. Epidemiological evidence reveals a markedly elevated risk of mild cognitive impairment and dementia among diabetic cohorts [3,4]. The chronic metabolic perturbations inherent to diabetes—predominantly persistent hyperglycemia, insulin resistance, and systemic inflammation—synergistically drive progressive neurodegeneration and structural cerebral alterations [5,6]. Chronic hyperglycemia drives excessive glucose flux through the polyol, advanced glycation end-product (AGE), protein kinase C (PKC) and hexosamine pathways, resulting in AGE accumulation, redox imbalance, mitochondrial dysfunction, and excessive reactive oxygen species (ROS) generation [7]. These metabolic disturbances activate AGE–RAGE-mediated oxidative and inflammatory signaling and impair cerebral insulin signaling, thereby disrupting neuronal metabolism and synaptic plasticity and ultimately promoting hippocampal neuronal injury and diabetes-associated cognitive impairment [8]. Despite extensive clinical and basic research evaluating insulin sensitizers, anti-inflammatory agents, and antioxidants, these interventions frequently yield limited efficacy and lack targeted molecular specificity, posing substantial challenges to the clinical management of DACI [9].

Emerging mechanistic studies increasingly identify ferroptosis as a principal pathological driver in the pathogenesis of DACI [10,11]. Ferroptosis is an iron-dependent form of regulated cell death biochemically characterized by excessive intracellular iron accumulation and lipid peroxidation [12,13]. In the diabetic brain, particularly within the highly vulnerable hippocampal region, chronic glucolipotoxicity disrupts iron homeostasis, depletes antioxidant defenses, and exacerbates the generation of ROS [14,15]. This toxic cascade inevitably culminates in lipid peroxidation-induced hippocampal neuronal death, fundamentally impairing synaptic plasticity and spatial memory [16]. Although the aforementioned studies have elucidated the involvement of ferroptosis, the current understanding of its regulatory mechanisms remains incomplete. Specifically, how it interacts with neuroprotective networks under the induction of disturbed glucose metabolism to affect DACI represents a critical mechanistic gap that restricts the development of effective therapeutic strategies. Peroxisome proliferator-activated receptor gamma (PPARγ) has long been recognized as a master regulator of lipid metabolism and insulin sensitivity; however, its neuroprotective potential, manifested through the active regulation of cell fate, is emerging as a focal point of recent research [17]. Crucially, as a pivotal upstream transcriptional activator of the nuclear factor erythroid 2-related factor 2 (Nrf2)/heme oxygenase-1 (HO-1) signaling axis, PPARγ orchestrates the primary cellular defense mechanisms against oxidative damage [18,19]. The activation of the PPARγ/Nrf2/HO-1 pathway can dynamically upregulate glutathione peroxidase 4 (GPX4), the core enzyme responsible for neutralizing lipid peroxides, thereby establishing robust resistance to ferroptosis [20]. While the previous literature has predominantly emphasized the individual functions of Nrf2 or GPX4, the present study conceptualizes the PPARγ/Nrf2/HO-1/GPX4 axis as a unified therapeutic target. This approach uniquely addresses both the underlying lipid metabolism disorders and the downstream execution of ferroptosis, providing a strategy for neuronal rescue.

Our previous research has demonstrated that TAC exerts a significant ameliorative effect on cognitive function in diabetic rats and exhibits considerable potential in inhibiting ferroptosis [21]. As a prominent traditional herbal medicine, rhizoma corydalis and its primary active constituent, TAC, have demonstrated remarkable neuroprotective, anti-inflammatory, and antioxidant activities across various preclinical models [22]. Despite these potent biological activities of TAC, its neuroprotective effects against DACI, especially the molecular mechanisms underlying ferroptosis regulation, remain largely unclear. Notably, multiple natural alkaloids have been identified as PPARγ modulators, which provides pharmacological evidence that PPARγ may serve as a targetable pathway for TAC-mediated cytoprotection. For instance, berberine suppresses NF-κB-dependent pyroptosis in a PPARγ-dependent manner to protect neurons against cerebral ischemia/reperfusion injury [23]. Moreover, certain alkaloids can activate PPARγ and alleviate inflammatory damage in microglia- and macrophage-associated models [24,25]. We hypothesize that TAC mitigates DACI by specifically binding to PPARγ and subsequently inhibiting hippocampal neuronal ferroptosis via the Nrf2/HO-1/GPX4 signaling pathway. To rigorously substantiate this hypothesis, we systematically integrated an in vivo diabetic animal model with an in vitro glucolipotoxic cell model. A notable strength of this study lies in the application of bioinformatics and single-cell RNA sequencing techniques, coupled with precise molecular docking and siRNA-mediated target knockdown. This multidimensional approach bridges system-level bioinformatic predictions with high-resolution cellular target validation, providing compelling evidence that TAC is a highly promising therapeutic agent against DACI.

## 2. Results

### 2.1. PPARγ Is Identified as a Key Target of TAC Regulating Ferroptosis in DACI PPARγ

Bioinformatics Analysis Results: The Venn diagram shows that DACI identifies 7956 relevant targets, while TAC identifies 57 relevant targets. Through mapping, 56 common targets shared between the disease and drug were identified (Figure 1A). Key targets were selected using the PPI network and the cytoHubba plugin for downstream functional enrichment analysis. The GO analysis yielded 385 entries, including 202 biological processes (BP), primarily involving the adenylate cyclase-activating adrenergic receptor signaling pathway, positive regulation of MAPK cascade, negative regulation of epinephrine secretion adenylate cyclase-inhibiting G protein-coupled acetylcholine receptor (Figure 1B); The Cell Composition (CC) section contains 39 entries, primarily involving the plasma membrane, postsynaptic membrane, glutamatergic synapse, and axon terminus; the Molecular Function (MF) section comprises 69 entries, mainly covering nuclear receptor activity, G protein-coupled acetylcholine receptor activity, enzyme binding, and alpha2-adrenergic receptor activity (Figure 1C); KEGG analysis identified 75 pathways, including neuroactive ligand–receptor interaction, the cAMP signaling pathway, the estrogen signaling pathway, cholinergic synapse, chemical carcinogenesis receptor activation, and the PPAR signaling pathway (Figure 1D). To further elucidate the signaling pathways involved in the core target genes, pathway matching was performed for the six core target genes, and a “core target gene pathways” network map was constructed. The results confirmed that PPARγ primarily participates in the PPAR signaling pathway, the Nrf2 signaling pathway, and the ferroptosis-related pathway (Figure 1E).

Molecular Docking Results: The top three compounds in the network—isocorypalmine, leonticine, and (s)-scoulerine—were docked with the core target proteins, ESR1, FOS, BDNF, CREB1, and PPARγ, in the PPI network. For the five targets, molecular docking analyses were performed on compounds with higher binding energies. The three-dimensional docking results demonstrated that isocorypalmine exhibited strong interactions with the target proteins CREB1, FOS, and PPARγ; (s)-scoulerine with CREB1 and PPARγ; and leonticine with ESR1 and PPARγ (Figure 1F). Table S1 lists the binding energy values of the three compounds shown. These findings confirm that TAC specifically binds to PPARγ, further establishing PPARγ as the target of TAC.

Overall, TAC may improve cognitive dysfunction in diabetic rats by regulating the Nrf2 signaling pathway and ferroptosis-related pathways through PPARγ.

### 2.2. Single-Cell Sequencing Confirms the Expression of PPARγ and Ferroptosis-Related Genes in Neurons

Single-cell RNA sequencing (scRNA-seq) analysis of the public dataset GSE201644 was performed to characterize the cell type-specific expression of key genes involved in the PPARγ/Nrf2 pathway and ferroptosis in the hippocampus. The results revealed that UMAP dimensionality reduction and clustering identified nine major cell populations in the hippocampus: microglia, astrocyte, oligodendrocyte, mature neuron, immature neuron, endothelial cell, VSMC, pericyte, and CPC. Cell type-specific marker gene expression profiles are presented in the heatmap (Figure 2A), and the distribution of each cell population is visualized in the UMAP plot (Figure 2B). Feature plots (Figure 2C) visualized the expression distribution of hub genes related to the PPARγ/Nrf2 pathway and ferroptosis (*Pparg, Hmox1, Nfe2l2, Gpx4*) across all cell clusters, and bubble plot analysis (Figure 2D) further revealed their distinct cell type-specific expression patterns. Notably, *Pparg* was predominantly expressed in microglia, astrocytes and neurons; *Hmox1* and *Nfe2l2* exhibited the highest expression in microglia and neurons; and *Gpx4* was widely detected in neurons and glial cells, with marked enrichment in mature neurons and oligodendrocytes. Overall, these scRNA-seq data demonstrate that genes involved in the PPARγ/Nrf2 pathway and ferroptosis exhibit distinct cell type-specific expression in the hippocampus, with neurons as the primary active cell populations, suggesting these cell types are key potential targets for TAC intervention in diabetic cognitive dysfunction.

### 2.3. The Ameliorative Effect of TAC on Diabetic Rats with Cognitive Dysfunction

During the 5-day Morris water maze (MWM) training period, model rats exhibited significantly prolonged escape latencies compared with normal controls, indicating impaired spatial learning. Treatment with medium- and high-dose TAC progressively shortened the escape latency, whereas metformin produced no significant improvement (Figure 3A). In the subsequent spatial probe test, diabetic rats spent less time in the target quadrant and crossed the original platform location fewer times than normal controls, demonstrating marked deficits in spatial memory. TAC treatment significantly improved both parameters in a dose-dependent manner, with the high-dose TAC group showing the strongest effect. Metformin also increased the time spent in the target quadrant, although its effect was less pronounced than that of high-dose TAC (Figure 3B–D). These findings indicate that TAC substantially mitigates diabetes-associated impairments in spatial learning and memory.

To characterize the metabolic changes accompanying these behavioral effects, fasting blood glucose (FBG) and body weight were monitored weekly throughout the 4-week treatment period. Both TAC and metformin significantly reduced FBG in diabetic rats (Figure 3E). Model rats exhibited a progressive loss of body weight, which was significantly attenuated by metformin but not by TAC (Figure 3F). Consistent with the hyperglycemic state, model rats also showed marked dyslipidemia, characterized by elevated serum total cholesterol (TC), triglycerides (TG), and low-density lipoprotein cholesterol (LDL-C), together with reduced high-density lipoprotein cholesterol (HDL-C) and insulin levels. Medium- and high-dose TAC significantly and dose-dependently improved these lipid abnormalities and increased serum insulin levels, whereas metformin primarily improved glycemic control and body-weight loss (Figure 3G,H).

Taken together, TAC produced coordinated improvements in both metabolic and cognitive phenotypes, although the magnitude of these effects varied across individual parameters. Notably, the cognitive benefits of TAC were evident together with improved glycemic and lipid homeostasis, whereas metformin, despite exerting clear metabolic effects, showed comparatively limited efficacy in the behavioral assessments. These findings suggest that the cognitive protection afforded by TAC may involve mechanisms beyond systemic metabolic improvement.

### 2.4. TAC Alleviates Hippocampal Neuron Damage and Inhibits Ferroptosis in Diabetic Rats

HE staining results: Light microscopic observations of HE staining showed that hippocampal neurons in the Normal group were neatly arranged with a clear structure, intact cell membranes, and uniform cytoplasm. In contrast, Model group neurons were disorganized, with clear pyknosis, nuclear condensation, cytoplasmic vacuolation, and extensive neuronal loss. Compared with the Model group, the TAC-mid and TAC-high groups significantly improved hippocampal neuronal arrangement, reduced pyknotic neurons, and alleviated neuronal loss—effects that were more pronounced in the TAC-high group. These findings indicate TAC mitigates hippocampal neuronal damage in diabetic rats (Figure 4A).

Transmission electron microscopy observations: TEM showed that Normal group hippocampal neurons had intact cell membranes, regular nuclei, and structurally intact mitochondria with well-preserved cristae. Model group neurons exhibited typical ferroptotic features: mitochondrial shrinkage and condensation, cristae loss or fragmentation, cytoplasmic vacuolation, and increased iron deposition, with no typical apoptotic morphological changes. Relative to the Model group, TAC-high group hippocampal neurons had significantly reduced mitochondrial shrinkage, largely intact cristae, and improved mitochondrial membrane integrity (Figure 4B). These results demonstrate that TAC improves mitochondrial morphology, inhibits ferroptosis, and protects neuronal function in diabetic rat hippocampi.

Perls staining and Fe^2+^ quantitative results: Perls staining revealed negligible blue precipitates in the Normal group brains, indicating low basal Fe^3+^ levels. A certain degree of blue deposition was observed in the model group, suggesting an accumulation of Fe^3+^. TAC-mid and TAC-high groups had significantly reduced blue precipitates compared with the Model group (Figure 4C,D). Quantitative assays showed that hippocampal Fe^2+^ levels were significantly elevated in the Model group vs. the Normal group; TAC-mid and TAC-high doses remarkably downregulated the Fe^2+^ content, with superior efficacy in the TAC-high group (Figure 4E). Quantitative results were consistent with qualitative staining, confirming that TAC inhibits Fe^2+^ overaccumulation. Peripheral blood Fe^2+^ levels showed a similar trend to hippocampal tissues (Figure 4F). Perls staining was used to provide histological evidence of iron deposition, whereas the quantitative iron assay was employed to further verify the changes in iron accumulation levels.

Oxidative stress indexes results: Compared with the Normal group, the Model group had significantly decreased hippocampal SOD activity, GSH content, and GSH-PX activity, along with significantly increased MDA and 4-HNE levels—indicating severe oxidative stress and lipid peroxidation in diabetic rats. Relative to the Model group, the TAC-mid and TAC-high groups significantly increased SOD activity, GSH content, and GSH-PX activity, while reducing MDA and 4-HNE levels (Figure 4G–K). These results confirm that TAC inhibits hippocampal oxidative stress and lipid peroxidation in diabetic rats.

### 2.5. Expression of Ferroptosis-Related Proteins

Immunohistochemistry (Figure 5A) and Western blot (Figure 5B) analysis were performed with β-actin serving as the internal reference. The results revealed that the expression levels of anti-ferroptotic GPX4 and xCT declined while the pro-ferroptotic protein ACSL4 increased in the Model group when compared with the Normal group. TAC intervention reversed the above alterations. Medium and high doses of TAC notably elevated the expression of GPX4 and xCT and decreased the level of ACSL4 relative to the Model group. Metformin treatment exerted a moderate regulatory effect on these proteins, yet its efficacy was inferior to that of high-dose TAC. Overall, TAC suppresses ferroptosis in the hippocampus of diabetic rats by modulating GPX4, xCT and ACSL4, and the therapeutic effect of TAC is stronger than metformin.

### 2.6. TAC Regulates Nrf2/HO-1/GPX4 Pathway Through PPARγ

Immunohistochemistry (Figure 6A) and Western blot (Figure 6B) analysis were performed with β-actin serving as the internal reference. The results revealed that the expression levels of PPARγ, Nrf2 and HO-1 were significantly reduced in the Model group when compared with the Normal group. TAC intervention reversed the above alterations. Medium and high doses of TAC notably elevated the expression of PPARγ, Nrf2 and HO-1 relative to the Model group. Overall, TAC activates the PPARγ/Nrf2/HO-1 pathway in the hippocampus of diabetic rats by upregulating PPARγ, Nrf2 and HO-1.

### 2.7. TAC Inhibits PA + HG-Induced Ferroptosis in HT22 Cells

Determination of Modeling Concentration and TAC-containing Serum Concentration: The CCK-8 assay showed the optimal PA + HG modeling condition and TAC-containing serum concentration when using the CCK-8 assay in HT22 cells. The combination of 2 mmol·L^−1^ PA and 25 mmol·L^−1^ HG reduced cell viability to approximately 70% of the control, which was selected as the modeling condition (Figure 7A). TAC-containing serum dose-dependently improved cell viability, with 14 μmol·L^−1^ identified as the optimal effective concentration (restoring viability to approximately 85% of the control) (Figure 7B,E). Fluorescence staining further confirmed that PA + HG significantly increased ROS and mitochondrial superoxide anion levels, which were markedly attenuated by 14 and 28 μmol·L^−1^ TAC-containing serum, with the strongest effect at 14 μmol·L^−1^ (Figure 7C,D,F). Western blot (Figure 7G) showed that PA + HG downregulated GPX4 and xCT, while upregulating ACSL4; TAC reversed these changes. Collectively, these data indicate that TAC-containing serum protects HT22 cells from PA + HG-induced ferroptosis-related injury.

PPARγ is the Key Target Mediating TAC’s Anti-Ferroptotic Effect: To verify the transfection efficiency of PPARγ siRNA, we detected PPARγ expression via q-PCR and Western blot (Figure 7H,I). Compared with the si-NC group, PPARγ mRNA and protein levels were significantly downregulated in the si-PPARγ group, confirming successful and effective gene silencing that met experimental requirements. We evaluated the effect of PPARγ silencing on TAC’s protective efficacy against PA + HG-induced ferroptosis. Western blot analysis (Figure 7G,I) showed that, compared with the si-NC + PA + HG + TAC group, silencing PPARγ significantly downregulated the expression of PPARγ, Nrf2, HO-1, and anti-ferroptotic GPX4, while upregulating pro-ferroptotic ACSL4. These findings indicate that PPARγ silencing abrogated the protective effect of TAC, confirming PPARγ as a key target mediating TAC’s inhibition of PA + HG-induced ferroptosis in HT22 cells.

## 3. Discussion

Diabetes-associated cognitive impairment (DACI) has emerged as one of the most severe chronic complications of diabetes mellitus; however, its pathogenesis and therapeutic strategies remain elusive, with us lacking an in-depth understanding of the core pathological mechanisms and targeted molecular interventions. Current clinical interventions primarily rely on therapeutic paradigms derived from mild cognitive impairment and Alzheimer’s disease. Nevertheless, numerous candidate drugs have failed to demonstrate significant efficacy in phase III clinical trials, underscoring the urgent need to identify novel mechanistic targets and therapeutic modalities [26]. In recent years, ferroptosis, a critical pathological mechanism linking metabolic dysregulation with neurodegeneration, has garnered increasing attention. However, whether traditional Chinese botanical extracts can alleviate DACI by modulating ferroptosis remains largely unknown. In the present study, we demonstrate for the first time that TAC significantly ameliorate cognitive impairment in diabetic rats by inhibiting ferroptosis via the activation of the PPARγ/Nrf2/HO-1/GPX4 signaling axis, a finding consistent with our previous reports. Importantly, our study integrates network pharmacology, molecular docking, in vivo animal models, single-cell transcriptomics, and in vitro validations, thereby establishing a robust and comprehensive mechanistic framework for TAC-mediated neuroprotection in DACI.

Accumulating evidence suggests that DACI is not merely a consequence of chronic hyperglycemia, but rather a multifactorial neurodegenerative process encompassing insulin resistance, oxidative stress, chronic neuroinflammation, mitochondrial dysfunction, and neuronal death [27]. Consistent with these observations, the present study successfully established a diabetic cognitive impairment model utilizing a prolonged high-fat and high-sugar diet combined with low-dose STZ administration. This modeling strategy more accurately simulates the progressive and insidious pathological features observed in patients with type 2 diabetes compared to acute hyperglycemia models. In addition to cognitive impairment and persistent hyperglycemia, diabetic rats exhibited a marked reduction in body weight, indicating that chronic glucose dysregulation was accompanied by substantial systemic metabolic disturbance. Behavioral analyses via the Morris water maze revealed significant deficits in spatial learning and memory among diabetic rats. The ability of TAC to mitigate this weight loss while reducing glucose indicates a partial restoration of systemic metabolic homeostasis. This metabolic correction may alleviate glucolipotoxic stress, thereby providing an important physiological basis for its downstream neuroprotective effects. TAC administration significantly abbreviated the escape latency and increased the frequency of platform crossings in a dose-dependent manner. These findings indicate that the neuroprotective efficacy of TAC extends beyond mere hypoglycemic actions. In order to comprehensively assess the overall therapeutic efficacy of TAC against DACI and T2DM-associated pathological changes, along with its potential for clinical translation, we chose metformin as a reference control. Notably, TAC outperformed metformin across multiple behavioral and molecular indices, suggesting that its underlying pharmacological mechanisms likely transcend fundamental metabolic correction.

Corydalis yanhusuo, a prominent traditional botanical medicine, has long been utilized to promote blood circulation, dissipate blood stasis, and alleviate pain. Emerging evidence increasingly highlights that its alkaloid constituents possess potent antioxidant, anti-inflammatory, and neuroprotective properties [22]. Network pharmacology analysis revealed that the PPAR signaling pathway, ferroptosis pathway, and Nrf2-related pathways are among the most highly enriched signaling cascades. Previous phytochemical profiling has identified isocorypalmine, leonticine, palmatine, and scoulerine as the principal bioactive components of TAC [28,29]. In the current investigation, molecular docking further elucidated strong binding affinities between these phytocompounds and PPARγ, substantiating the hypothesis that PPARγ serves as a critical pharmacological target of TAC. Furthermore, single-cell transcriptomic analysis revealed cell-type-specific expression of the PPARγ/Nrf2/HO-1/GPX4 pathway in the hippocampus, with neurons, microglia, and astrocytes representing the major cell populations expressing these pathway-related genes. This finding supports the potential involvement of PPARγ in the regulation of lipid metabolism, oxidative stress, mitochondrial homeostasis, and neuronal ferroptosis. However, these pathways are not neuron-specific. *PPARγ*, *Nfe2l2*, and *Hmox1* are also expressed in glial cells, including microglia and astrocytes, where these pathways participate in the regulation of inflammatory and oxidative responses [30]. In particular, Nrf2 activation in astrocytes can enhance antioxidant defenses and confer non-cell-autonomous protection to neighboring neurons, while microglial Nrf2/HO-1 signaling may attenuate neuroinflammation and oxidative stress [31]. Therefore, TAC-mediated neuroprotection may involve both neuronal signaling and glia-mediated effects. Nevertheless, the present study did not determine the cell-specific effects of TAC on microglia or astrocytes, and their contribution requires further investigation. Notably, although the in vivo experiments were conducted in an STZ-induced diabetic rat model, the bioinformatics analysis was based on a db/db mouse scRNA-seq dataset. This dataset was used only to characterize cell-type-specific gene expression and was not intended to model TAC responses. Future studies using rat-derived single-cell transcriptomic data will be valuable for further validating these mechanisms.

Recent studies have underscored ferroptosis as a central driver of diabetic complications, particularly DACI [15]. Ferroptosis is biochemically characterized by intracellular iron overload and massive lipid peroxidation, ultimately culminating in catastrophic membrane permeabilization and cell death [11]. Under conditions of chronic hyperglycemia, the convergence of glycation reactions, insulin resistance, and inflammatory activation disrupts systemic and local iron homeostasis, precipitating the accumulation of Fe^2+^ within neural tissues [32]. This excessive iron subsequently catalyzes the Fenton reaction, generating voluminous amounts of ROS and triggering a deleterious lipid peroxidation cascade and ensuing neuronal injury [33]. Congruent with prior investigations, our morphological and biochemical analyses revealed severe iron deposition in the hippocampi of diabetic rats, accompanied by elevated levels of malondialdehyde (MDA) and 4-hydroxynonenal (4-HNE), alongside diminished superoxide dismutase (SOD) activity. Crucially, TAC administration significantly mitigated iron accumulation and attenuated oxidative stress, indicating that the abrogation of ferroptosis constitutes a primary mechanism underlying its neuroprotective efficacy. Canonical morphological alterations associated with ferroptosis include mitochondrial shrinkage, reduction or vanishing of cristae, and increased outer membrane density; consequently, mitochondria are widely acknowledged as the principal organelles compromised during ferroptotic stress [10]. Transmission electron microscopy (TEM) evaluations in this study unequivocally revealed these ultrastructural hallmarks within diabetic hippocampal neurons, whereas TAC treatment robustly restored mitochondrial integrity. These structural findings provide compelling evidence that TAC suppresses ferroptosis-mediated neuronal demise. Interestingly, contemporary research further posits that ferroptosis functions not merely as a terminal downstream event but as an active amplifier of neuroinflammation and synaptic dysfunction in the diabetic brain, thereby accelerating cognitive decline [34]. Our results align strongly with this evolving paradigm. Currently, the xCT-GSH-GPX4 axis is recognized as the core endogenous defense system safeguarding against ferroptosis [35]. The cystine/glutamate antiporter (xCT) maintains intracellular cysteine availability for glutathione (GSH) biosynthesis, while GPX4 reduces highly toxic lipid hydroperoxides to non-toxic lipid alcohols. Dysfunction within this antioxidant axis is considered a definitive hallmark of ferroptosis. In our investigation, diabetic rats exhibited significantly downregulated expressions of GPX4 and xCT, coupled with upregulated ACSL4 levels, reflecting profound ferroptosis activation. TAC markedly restored the expression levels of GPX4 and xCT while concurrently repressing ACSL4, suggesting that TAC ameliorates ferroptosis by re-establishing intracellular redox homeostasis and facilitating lipid peroxide detoxification. Analogous protective mechanisms have recently been documented in models of diabetic cardiomyopathy and ischemic neuronal injury, wherein the activation of GPX4-dependent pathways effectively circumvented ferroptotic cell death [36,37].

An expanding body of evidence highlights the robust antioxidant and anti-inflammatory properties of PPARγ across a spectrum of metabolic and neurodegenerative disorders [38]. Furthermore, recent studies propose that PPARγ synergizes with Nrf2 to suppress ferroptosis by driving GPX4 transcription and maintaining lipid homeostasis [39]. In the present study, both IHC and Western blotting analyses demonstrated a significant downregulation of PPARγ expression in the diabetic hippocampus, which was robustly reversed following TAC treatment. These dynamic alterations paralleled changes in Nrf2 and HO-1 expression levels, implying a tightly coordinated mechanistic synergy. Nrf2 is canonically recognized as the master transcriptional regulator of cellular antioxidant defense networks. Upon oxidative insult, Nrf2 dissociates from its repressor Keap1 and translocates into the nucleus, where it binds to antioxidant response elements (AREs) to activate downstream cytoprotective genes, including HO-1, GPX4, and xCT [40]. The activation of the Nrf2/HO-1 pathway has been extensively documented to abrogate ferroptosis in diverse metabolic and neurodegenerative paradigms [41,42]. TAC significantly upregulated the expression levels of Nrf2 and HO-1 in the diabetic hippocampus, indicating that TAC enhances endogenous antioxidant buffering capacity via PPARγ-mediated Nrf2 activation. This highlights the unique pharmacological advantage of TAC in modulating oxidative stress-associated neuronal injury. To definitively ascertain the causal role of PPARγ, we employed siRNA-mediated targeted gene silencing in HT22 cells. PPARγ knockdown nearly abolished the protective effects of TAC against palmitic acid and high glucose (PA + HG)-induced ferroptosis, as evidenced by the concurrent abrogation of Nrf2, HO-1, and GPX4 upregulation, alongside the persistence of elevated ACSL4 levels. These in vitro findings definitively validate the role of PPARγ as an indispensable molecular switch dictating TAC-mediated ferroptosis inhibition. Mechanistically, PPARγ likely facilitates the transcriptional activity of Nrf2 and bolsters GPX4-mediated lipid peroxide detoxification, thereby preserving neuronal viability under lipotoxic and glucotoxic stress conditions.

To fully appreciate the therapeutic significance of TAC, it is essential to contextualize our findings within the broader landscape of alkaloid-mediated pharmacology. Similar to our findings with TAC, previous studies have shown that several natural alkaloids protect neurons or other cells by modulating overlapping pathways, either by upregulating PPARγ to suppress inflammatory and oxidative injury [23,25,43] or by activating the Nrf2-dependent antioxidant system and enhancing the SLC7A11/GSH/GPX4 axis to inhibit ferroptosis [44,45]. However, while prior investigations generally evaluated these upstream metabolic regulators (PPARγ) and downstream cell death executioners (ferroptosis) as distinct mechanisms, our findings demonstrate that TAC may integrate these pathways into a unified protective cascade involving the PPARγ/Nrf2/HO-1/GPX4 axis. This indicates that TAC may possess the capacity to simultaneously alleviate systemic metabolic stress and inhibit neuronal ferroptosis, which may contribute to its therapeutic potential in DACI.

Nevertheless, several limitations should be considered when interpreting the present findings. First, TAC is a multicomponent alkaloid mixture, and although the present study supports its overall protective effects against diabetic cognitive impairment, the individual contributions of its constituent alkaloids and their potential synergistic interactions remain to be determined. Second, although our findings support a role for ferroptosis in TAC-mediated neuroprotection, its crosstalk with neuroinflammation, autophagy, mitochondrial dysfunction, and other pathways involved in diabetic cognitive impairment warrants further investigation. In addition, diabetic cognitive impairment is a multifactorial process involving diverse pathological mechanisms, including AGEs/RAGE signaling, synaptic dysfunction, cholinergic alterations, and brain insulin resistance, which were beyond the scope of the present study. Thus, the contribution of these pathways to the effects of TAC cannot be excluded. While our in vitro siRNA experiments established a definitive causal relationship at the cellular level, the absence of an in vivo pharmacological positive control (such as thiazolidinediones) limits our ability to conduct a direct comparative efficacy analysis of PPAR-γ activation. Furthermore, the lack of direct comparison with established cognitive-enhancing agents restricts our capacity to fully contextualize the magnitude and mechanistic advantages of TAC-mediated cognitive protection within the landscape of currently available pharmacological interventions.

## 4. Material and Methods

### 4.1. Bioinformatics

#### 4.1.1. Bioinformatics Analysis

The structure of TAC was obtained using the Traditional Chinese Medicine System Pharmacology Database and Analysis Platform (https://tcmsp-e.com/, accessed on 15 March 2026) and the HERB database (http://herb.ac.cn/, accessed on 15 March 2026), with the structural file saved in MOL2 format. This file was uploaded to Swiss Target Prediction (http://www.swisstargetprediction.ch/, accessed on 15 March 2026) to predict the target of TAC. Targets obtained from different databases were merged and deduplicated to identify potential targets for TAC. By searching using the terms “diabetic cognitive dysfunction” and “Homo sapiens,” targets related to diabetic cognitive dysfunction were retrieved from the Genecards database (https://www.genecards.org/, accessed on 15 March 2026) and the National Center for Biotechnology Information database (NCBI, https://www.ncbi.nlm.nih.gov/, accessed on 15 March 2026). The targets obtained from these two databases were further merged and deduplicated using Veen 2.1 to identify disease targets associated with diabetic cognitive dysfunction.

In the Cytoscape 3.9.1 platform, the intersecting target data were imported to construct a thematic relationship network centered on “Traditional Chinese Medicine—Active Components—Targets,” with node degree values analyzed for screening key active components. Subsequently, the genetic information of the intersecting targets was imported into the STRING database (https://string-db.org/, accessed on 15 March 2026), with species set to Homo sapiens, the minimum interaction confidence threshold adjusted to the highest value (>0.9), and other parameters maintained at default settings. The results were saved as tsv format files. Following these steps, the CytoHubba and MCODE functional modules in Cytoscape were utilized to conduct in-depth analysis of the core targets, identifying key node gene sets and constructing a protein–protein interaction (PPI) network map.

Using the DAVID database (https://david.ncifcrf.gov/tools.jsp, accessed on 15 March 2026), we conducted Gene Ontology (GO) analysis and KEGG enrichment analysis on the overlapping targets, examining them at three levels: cellular components (CC), biological processes (BP), and molecular functions (MF). Enrichment significance was evaluated using a hypergeometric test, and *p* values were corrected for multiple testing using the Benjamini–Hochberg method. GO terms with an adjusted *p* value (FDR) < 0.05 were considered statistically significant. We systematically identified significantly enriched pathways and generated intuitive visualizations using R language.

#### 4.1.2. Molecular Docking

The crystal structures of the primary targets of the total alkaloids from Corydalis yanhusuo were obtained from the RCSB Protein Database (http://www.pdb.org/, accessed on 15 March 2026). The three-dimensional coordinates of the protein activity pockets were determined using PyMOL software 2.5.0. The structural file of the protein receptor was processed by removing non-protein components (including ligands and solvent molecules) and saved in PDB format. Subsequently, the structure was optimized using AutoDock Vina 1.2.7, primarily involving optimization of amino acid side-chain conformations and the addition of hydrogen atoms [46]. The processed protein receptor file was then converted to the PDBqt format for subsequent analysis. Energy minimization calculations for the ICA molecule were performed using Chem3D software 20.0, with the results saved in MOL2 format. The molecular structure was imported into AutoDock Tools 1.5.7, and after necessary format conversions, the results were exported as a PDBqt file for ligand representation. Following these preparatory steps, molecular docking simulations were executed using Autodock Vina, and the results were visualized with PyMOL 3.0.3, ultimately generating and presenting the three-dimensional structural map of the molecular docking [47]. The two-dimensional interaction diagram was generated using Discovery Studio.

#### 4.1.3. Single-Cell Sequencing Analysis

Single-cell transcriptome data related to the diabetic hippocampus (GSE201644; https://www.ncbi.nlm.nih.gov/geo/, accessed on 15 March 2026) were retrieved from the public GEO database. This dataset contains hippocampal Single-cell transcriptomic profiles from db/db diabetic mice and db/m control mice. The dataset was processed using Seurat V4 [48] in the R language, employing the Create Seurat Object function with parameters set to min.cells = 3 and min.features = 200. Cells with fewer than 200 genes were removed, while genes expressed in at least three cells were retained; the cell filtration threshold was set at MT < 15%. After data normalization, dimensionality reduction was performed using the RunTSNE and RunUMAP functions for t-SNE and UMAP visualization, respectively. Cell cluster annotations were generated by identifying marker genes specific to different cell types and combining them with automatic cell type labeling performed by the ScType software version3. The top 2000 variable genes were identified using the FindVariableFeatures function. Dimensionality reduction was achieved through principal component analysis (PCA) using the RunPCA function, with the ElbowPlot function employed to determine the optimal number of principal components. The clustree package was utilized to determine the optimal resolution [49].

### 4.2. Experimental Animals

The experiment employed SPF-grade male SD rats, aged 8 weeks, with a body weight range of 180 ± 20 g. A total of 48 rats were provided by Changsheng Biotechnology Co., Ltd. (Changchun, China). The relevant experimental approval number is SCXK (Liao) 2020-0001. The animal experiments were approved by the Experimental Animal Welfare and Ethics Committee of Heilongjiang University of Chinese Medicine, with project number: 2023112907. The rats were allowed free access to food and water under conditions of a temperature of 20–24 °C and a relative humidity of 50–60%. All animals were maintained under a 12 h light/12 h dark cycle and provided with sterilized standard rodent chow and sterile drinking water ad libitum. Strict pre-defined humane endpoints were established for this animal study, including sustained body weight loss exceeding 20% of the initial baseline value, progressive lethargy, difficulty in feeding and movement, self-mutilation or severe uncontrollable infection. Animals that met any humane endpoint criterion were immediately euthanized via carbon dioxide inhalation in accordance with institutional ethical standards.

### 4.3. Experimental Reagents and Instruments

#### 4.3.1. Experimental Reagents

According to the preliminary methodology developed by the research team for TAC preparation, the specific extraction procedure employed was acid-water percolation. The endpoint of percolation was determined using alkaloid precipitation reagents, with Corydalis etinolide serving as the standard substance. The TAC content was measured employing the acid dye colorimetric method. After comprehensive evaluation of all factors, the optimal percolation volume was determined to be 12 times the volume required to achieve the desired medicinal quality, yielding a TAC extraction efficiency of 85% [21,50]. The materials used included a high-fat and high-sugar diet (78.8% basal feed, 15% lard, 1% cholesterol, 5% sucrose, 0.2% sodium cholate). Streptozotocin (S6050F) and metformin (MC10640) were purchased from Beijing Bo’ao Tuoda (Beijing, China) and Liaoning Meilune (Dalian, China), respectively. Assay kits for lipid metabolism, oxidative stress, and iron metabolism (A110-1-1, A111-1-1, A112-1-1, A113-1-1, A001-3-2, A003-1-2, A006-2-1, A005-1-2, A039-2-1, and A039-1-1), as well as a rabbit two-step detection kit (A003-1-2), were obtained from Nanjing Jiancheng Bioengineering Institute (Nanjing, China). Beijing Aotuoda (Beijing, China) supplied the insulin kit (TOPEL02853), 4-HNE kit (TOPEL30239), and PVDF membranes (ISEQ00010). Protein markers (C05-09002) and antibodies for HO-1 (bs-23397R), GAPDH (bs-8789R), and β-actin (bs-1165R) were acquired from Beijing Biosynthesis (Beijing, China). The BCA protein assay kit (MA0082-2) and ECL substrate (MA0186) were purchased from Dalian Meilune (Dalian, China), whereas PAGE gel kits (PG112) were obtained from Shanghai YaMei (Shanghai, China). HRP-conjugated goat anti-rabbit IgG (PV-6000) was purchased from Beijing ZSGB-BIO (Beijing, China). Antibodies against GPX4 (FNab03622) and PPARγ (FNab06661) were obtained from Wuhan Feien (Wuhan, China), the antibody against xCT (GW083698) was obtained from Wuhan ABclonal (Wuhan, China), and antibodies against Nrf2 (BF8017) and ACSL4 (DF12141) were obtained from Affinity Biosciences (Cincinnati, OH, USA). The CCK-8 kit (K1018) was purchased from APExBIO (Houston, TX, USA). The ferroptosis detection kit (C1190S) was obtained from Shanghai Beyotime (Shanghai, China). HT22 cell culture medium (TCM-G821), DMEM (BL304A), and palmitic acid (R43697-2) were purchased from HyCyte (Suzhou, China), Beijing Lanjieke (Beijing, China), and Shanghai Yuanye (Shanghai, China), respectively. TransZol Up (ET111) and cDNA SuperMix (AT311) were purchased from TransGen Biotech (Beijing, China); qPCR Master Mix (Q711) was obtained from Vazyme (Nanjing, China); and PPARγ siRNA/CALNP™ RNAi (DN001) was obtained from Shanghai Duona (Shanghai, China).

#### 4.3.2. Experimental Instruments

The main instruments used in this study were as follows: a blood glucose meter (Accu-Chek Instant S, Roche Diagnostics, Basel, Switzerland, a Morris water maze video tracking system (Xinruan Information Technology Co., Ltd., Shanghai, China), a real-time quantitative PCR system (Mx3000P, Agilent Technologies, Santa Clara, CA, USA), Benchtop high-speed refrigerated centrifuges (TGL16, Yingtai Instrument Co., Ltd., Changsha, China), a microplate reader (DNM-9602, Perlong New Technology Co., Ltd., Beijing, China), an upright fluorescence microscope (FR-4A, Optical Instrument Factory, Shanghai, China) equipped with a microscopic imaging system (Motic BA400, USA Motic Inc.), a tissue microtome (YD-1508B, Yidi Medical Appliance Co., Jinhua, China), a CO_2_ incubator (CLM-170/240L, Esco Lifesciences, Shanghai, China), an electrophoresis apparatus including a power supply (EPS-300), electrophoresis tank (VE-186), and transfer tank (VE-180B), and a gel imaging system (Tanon-4600), all purchased from Tanon Technology Co., Ltd. (Shanghai, China). 

### 4.4. Experimental Methods

#### 4.4.1. Establishment of Diabetic Rat Model

Following a one-week acclimation period, the rats were maintained on a high-fat and high-sugar diet for 16 consecutive weeks. At weeks 17 and 18, streptozotocin (STZ, 35 mg·kg^−1^) was administered via intraperitoneal injection, with a one-week interval between the two doses. Seven days following the final injection, the successful establishment of the diabetes-associated cognitive impairment model was validated in 40 rats through fasting blood glucose (FBG) measurements and Morris water maze (MWM) testing. Specifically, only rats with a blood glucose level (FBG) of ≥16.7 mmol·L^−1^ and showing a significantly prolonged escape latency compared to the healthy control group in the MWM test were included in this study. The initial 48 rat cohort was reduced to 40 rats. Rats that died during the modeling stage or failed to meet the threshold requirements for blood glucose and behavior were excluded.

The sample sizes for each group were determined based on our previous experimental data and published results from similar pharmacological studies. A priori statistical power analysis was conducted using the G*Power 3.1 software. Specifically, 8 rats per group were arranged for behavioral tests and biochemical detection, while 3 independent biological replicates (3 different rats per group) were used for molecular assays including Western blot and immunohistochemistry. Post hoc power analysis confirmed that the current sample size achieved sufficient statistical power (>0.80) to detect significant intergroup differences with stable and repeatable results. For all experimental procedures, strict blinding protocols were implemented. The researcher responsible for Morris water maze behavioral testing, histological staining evaluation and quantitative data analysis was completely blinded to the group allocation of animals. All grouping information was unlocked only after the completion of all data collection and quantitative analysis to avoid subjective observation bias.

These 40 confirmed model rats were subsequently randomized into five groups (n = 8 per group) using a computer-generated random number sequence created in SPSS 26.0 software: the Model group, the Metformin-treated group (Met, 40mg·kg^−1^·d^−1^), and three TAC-treated groups at varying dosages (TAC-low: 7 mg·kg^−1^·d^−1^; TAC-mid: 11 mg·kg^−1^·d^−1^; TAC-high: 14 mg·kg^−1^·d^−1^). The TAC doses employed in this study were established through empirical optimization across a low, medium, and high range, aiming to evaluate the dose–response relationship and the effective therapeutic window. The 11 mg·kg^−1^ dose demonstrated significant biological efficacy in our preliminary experiments, prompting its selection as the medium-dose group. Additionally, this dosing rationale is well-supported by previous research [21]. Freshly formulated TAC and Met, utilizing physiological saline as the vehicle, were administered daily via oral gavage for four consecutive weeks. Concurrently, an additional eight randomly selected rats were allocated to the Control group and gavaged with an equivalent volume of physiological saline. FBG levels and body weight fluctuations were monitored weekly throughout the entire experimental duration. Following the intervention, cognitive performance was evaluated utilizing the MWM test. Two days post-behavioral assessment, the subjects were euthanized to harvest tissues for subsequent downstream analyses. Importantly, all subjects survived the entire study duration, and no animals or data points were excluded post hoc from any analyses.

#### 4.4.2. Preparation of TAC-Containing Serum

SD rats were orally gavaged with TAC (14mg·kg^−1^·d^−1^) for 28 consecutive days. At 1 h after the last gavage, abdominal aorta blood was collected under anesthesia, then centrifuged at 3000 r·min^−1^ for 15 min at 4 °C, and the upper serum was taken. The serum was inactivated in a 56 °C water bath for 30 min, filtered and sterilized with a 0.22 μm filter membrane, and stored at −80 °C for later use.

#### 4.4.3. Morris Water Maze Test

The rats underwent the Morris water maze test [51]: (1) Orientation navigation test: Each group of rats received training sessions spaced one hour apart. The rats were placed in the water facing the pool wall and released from four distinct quadrant markers. The time required to locate the platform within 60 s was recorded as the latency period. For rats that failed to complete the task within 60 s, they were returned to the platform for 10 s before being removed from the maze; their latency period was recorded as 60 s. The final result was the average of four measurements, and this training period lasted four days. (2) Spatial exploration test: On the fifth day, the platform was removed. Under unchanged conditions, the rats were ensured to remain in the opposite quadrant. Two key metrics were recorded: first, the time spent searching for the escape platform; second, the number of times the rats passed through the designated platform location during this phase.

#### 4.4.4. Detection of Serum Indexes

Upon completion of the Morris water maze assessment, all rats were fasted for 12 h. Subsequently, blood samples were harvested from the abdominal aorta. The blood was centrifuged at 3000 r·min^−1^ for 15 min at 4 °C to separate serum. Serum total cholesterol (TC), triglyceride (TG), high-density lipoprotein cholesterol (HDL-C), low-density lipoprotein cholesterol (LDL-C) and serum insulin levels were detected by an ELISA kit according to the kit instructions.

#### 4.4.5. HE Staining to Evaluate Hippocampal Neuron Damage 

After blood collection, rats were perfused with normal saline and 4% paraformaldehyde through the heart successively. Brain tissues were fixed in 4% paraformaldehyde for 24 h, followed by gradient ethanol dehydration, paraffin embedding, and sectioning into 5 μm thick slices. HE staining was performed according to the kit instructions: dewaxing with xylene, rehydration with gradient ethanol, staining with hematoxylin for 5 min, differentiation with 1% hydrochloric acid ethanol, bluing with tap water, staining with eosin for 3 min, dehydration with gradient ethanol, transparency with xylene, and sealing with neutral gum. The sections were observed under a light microscope, and 3 visual fields were randomly selected from each section to observe the morphological changes in hippocampal neurons. The damage degree of hippocampal neurons was evaluated by the number of normal neurons and the degree of pyknosis.

#### 4.4.6. Transmission Electron Microscopy to Observe Mitochondrial Changes in Hippocampal Neurons

The hippocampal tissue was cut into 1 mm^3^ small pieces, fixed in 2.5% glutaraldehyde at 4 °C for 24 h, rinsed with 0.1 mol·L^−1^ phosphate buffer (PBS) 3 times, fixed with 1% osmic acid for 1.5 h, rinsed with PBS 3 times, dehydrated with gradient ethanol, embedded in epoxy resin, polymerized at 60 °C for 48 h, cut into 50–70 nm ultra-thin sections, stained with uranyl acetate and lead citrate, and observed under a transmission electron microscope. The morphological changes in mitochondria (such as mitochondrial swelling, cristae rupture, membrane integrity) were observed and photographed.

#### 4.4.7. Prussian Blue Staining and Fe^2+^ Quantitative Detection

Prussian blue staining was performed to detect the distribution of Fe^2+^ in brain tissues. The paraffin sections were dewaxed, rehydrated, incubated with Prussian blue staining solution at 37 °C for 30 min, rinsed with tap water, counterstained with nuclear fast red for 5 min, dehydrated, transparent and sealed. The sections were observed under a light microscope, and the blue precipitate was the positive signal of Fe^2+^. Image-Pro Plus software 6.0 was used to quantify the positive area ratio of Fe^2+^.

The Fe^2+^ levels in hippocampal tissue and serum were quantitatively detected by an iron assay kit. The hippocampal tissue was homogenized with normal saline to prepare 10% tissue homogenate, then centrifuged at 3000 r·min^−1^ for 15 min at 4 °C, and the supernatant was taken. We took an appropriate amount of prepared rat serum sample. The Fe^2+^ level was detected according to the kit instructions, and the absorbance was measured at 593 nm by the ELISA reader. The Fe^2+^ content was calculated according to the standard curve. The Prussian blue method was validated for quantitative accuracy by referring to the linear regression method reported in relevant studies.

#### 4.4.8. Detection of Oxidative Stress Indexes in Hippocampus

The hippocampal tissue homogenate was prepared as described in Section 4.4.7. The contents and activities of SOD, MDA, GSH, GSH-PX and 4-HNE in the homogenate supernatant were detected according to the kit instructions. The absorbance was measured by the ELISA reader, and the corresponding indexes were calculated according to the standard curve.

#### 4.4.9. Immunohistochemistry Detection of Protein Expression

The paraffin sections were dewaxed, rehydrated, antigen retrieved with citrate buffer (pH 6.0) by high pressure, cooled to room temperature, and rinsed with PBS 3 times. The sections were incubated with 3% H_2_O_2_ at room temperature for 10 min to block endogenous peroxidase, and rinsed with PBS 3 times. Then, the sections were blocked with 5% BSA at 37 °C for 30 min, and incubated with primary antibodies (GPX4, xCT, ACSL4, PPARγ, Nrf2, HO-1, 1:100 dilution) at 4 °C overnight. The next day, the sections were rinsed with PBS 3 times, incubated with HRP-labeled secondary antibody (1:200 dilution) at 37 °C for 1 h, and rinsed with PBS 3 times. DAB chromogenic solution was added for chromogenic reaction, hematoxylin counterstaining, differentiation, bluing, dehydration, transparent and sealing. The sections were observed under a light microscope, and 3 visual fields were randomly selected from each section. Image-Pro Plus 6.0 software was used to quantify the average optical density of positive signals, which reflected the protein expression level.

#### 4.4.10. Western Blot Detection of Protein Expression

The hippocampal tissues or HT22 cells were lysed with RIPA lysis buffer (containing PMSF) on ice for 30 min, then centrifuged at 12,000 r·min^−1^ for 15 min at 4 °C, and the supernatant was taken as total protein. The protein concentration was determined by the BCA method. The protein samples were mixed with loading buffer, denatured at 95 °C for 10 min, separated by SDS-PAGE electrophoresis, and transferred to PVDF membrane. The membrane was blocked with 5% skimmed milk at room temperature for 1 h, and incubated with primary antibodies (GPX4, xCT, ACSL4, PPARγ, Nrf2, HO-1, GAPDH, β-actin, 1:1000 dilution) at 4 °C overnight. The next day, the membrane was rinsed with TBST 3 times, incubated with HRP-labeled secondary antibody (1:5000 dilution) at room temperature for 1 h, and rinsed with TBST 3 times. ECL chemiluminescence reagent was added, and the protein bands were imaged by a chemiluminescence imaging system. ImageJ software 6.0 was used to quantify the gray value of protein bands, and the relative expression level of target protein was calculated with β-actin or GAPDH as internal references.

#### 4.4.11. Cell Culture and Modeling

The HT22 cell line, an immortalized mouse hippocampal neuronal cell line derived from the HT-4 subclone, was obtained from HyCyte (Cat. No. TCM-C821, Suzhou, China). HT22 cells were cultured in their complete medium within a cell incubator containing 5% CO_2_ at 37 °C. Passaging was performed when the cell fusion rate reached 70–80%.

The ferroptosis model in HT22 cells was established using PA + HG. PA was dissolved in anhydrous ethanol to prepare a stock solution of 100 mmol·L^−1^, which was then diluted to various concentrations (1, 2, 3, 4, 5 mmol L^−1^) using a constant high-glucose culture medium containing 25 mmol·L^−1^ glucose. HT22 cells were seeded in 96-well plates and cultured until cell adhesion was achieved, after which they were treated with different concentrations of PA + HG for 24 h. Cell viability was assessed using the CCK-8 assay to determine the optimal PA + HG concentration required for model establishment.

#### 4.4.12. Determination of TAC-Containing Serum Concentration

HT22 cells were inoculated into 96-well plates, and after adherence, they were treated with PA + HG (optimal concentration) and different concentrations of TAC-containing serum (7, 14, 28, 56 mmol·L^−1^) for 24 h. The CCK-8 assay was used to detect cell viability, and the optimal TAC-containing serum concentration (the concentration that can significantly improve cell viability) was determined. At the same time, a RhoNox-6/CM-H2DCFDA ferroptosis fluorescence detection kit was used to detect the fluorescence intensity of Fe^2+^ and ROS in cells, and the inhibitory effect of TAC-containing serum on ferroptosis was verified.

#### 4.4.13. siRNA Transfection

HT22 cells were transfected with double-stranded siRNAs targeting PPARγ and control siRNA using the CALNP™ RNAi in vitro transfection reagent according to the manufacturer’s instructions. The sequences of the PPARγ siRNA were *5′-CAACAGGCCUCAUGAAGAATT-3′* and *5′-UUCUUCAUGAGGCCUGUUGTT-3′*, while those of the negative control siRNA were *5′-UUCUCCGAACGUGUCACGUTT-3′* and *5′-ACGUGACACGUUCGGAGAATT-3′*. Transfection efficiency was assessed by q-PCR.

#### 4.4.14. q-PCR Detection of mRNA Expression

Total cellular RNA was extracted using the TransZol Up reagent. cDNA was amplified using the PerfectStart^®^ Green qPCR SuperMix on the Bio-Rad CFX Manager 3.1 system, followed by reverse transcription with the EasyScript^®^ First-Stage gDNA Removal and cDNA Synthesis SuperMix Kit. mRNA levels were determined by the 2^−ΔΔCt^ method, with β-actin serving as the reference gene. 

#### 4.4.15. Statistical Analysis

All experimental data were expressed as mean ± SD and analyzed by SPSS 26.0 software. Prior to statistical analysis, the Shapiro–Wilk test was used to verify the normal distribution of data, and Levene’s test was applied to evaluate the homogeneity of variance to meet the assumptions of one-way analysis of variance (ANOVA). Comparison between multiple groups was performed by one-way ANOVA, and pairwise comparisons were performed using Tukey’s post hoc test to strictly correct for multiple comparisons. For data with uneven variance, Welch’s ANOVA and the Games–Howell post hoc test were adopted alternatively. *p* < 0.05 was considered statistically significant.

## 5. Conclusions

The present study definitively demonstrates that TAC significantly ameliorates diabetes-associated cognitive impairment by inhibiting neuronal ferroptosis via the targeted activation of the PPARγ/Nrf2/HO-1/GPX4 signaling cascade. Our findings not only reinforce the conceptual framework that ferroptosis is a core pathological driver of DACI but also provide a compelling experimental rationale for the advancement of TAC as a promising multi-target natural therapeutic agent for diabetic neurodegeneration.

## Figures and Tables

**Figure 1:**
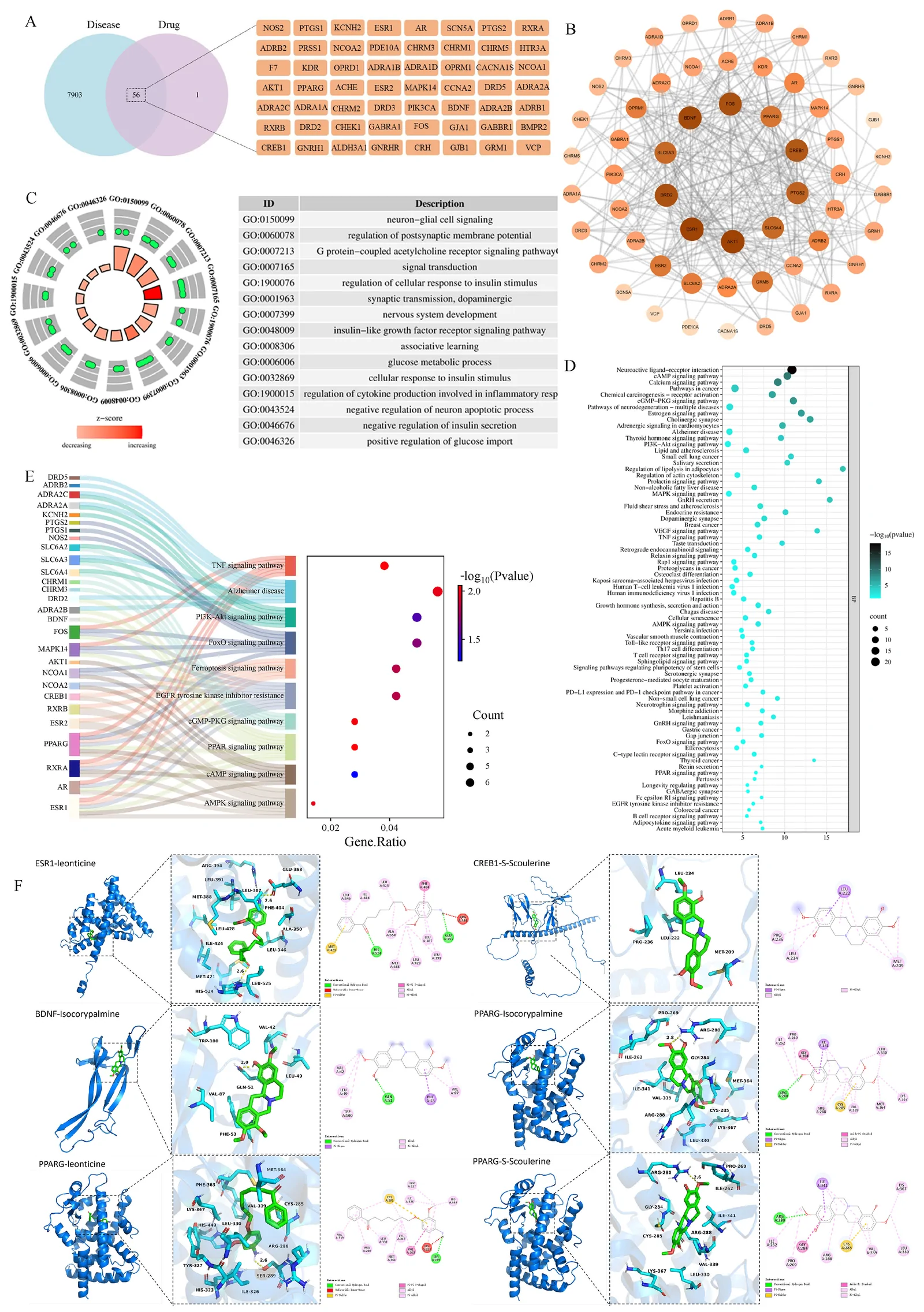
Bioinformatics analysis and molecular docking of TAC against diabetic cognitive dysfunction. (**A**) Venn diagram of overlapping targets between TAC and diabetic cognitive dysfunction (56 common targets). (**B**) PPI network of the 56 common targets. (**C**) GO enrichment analysis (BP, CC, MF) of the common targets. (**D**) KEGG pathway enrichment analysis of the common targets. (**E**) Sankey diagram of core hub genes and their associated signaling pathways. (**F**) Molecular docking of main TAC compounds with core target proteins.

**Figure 2:**
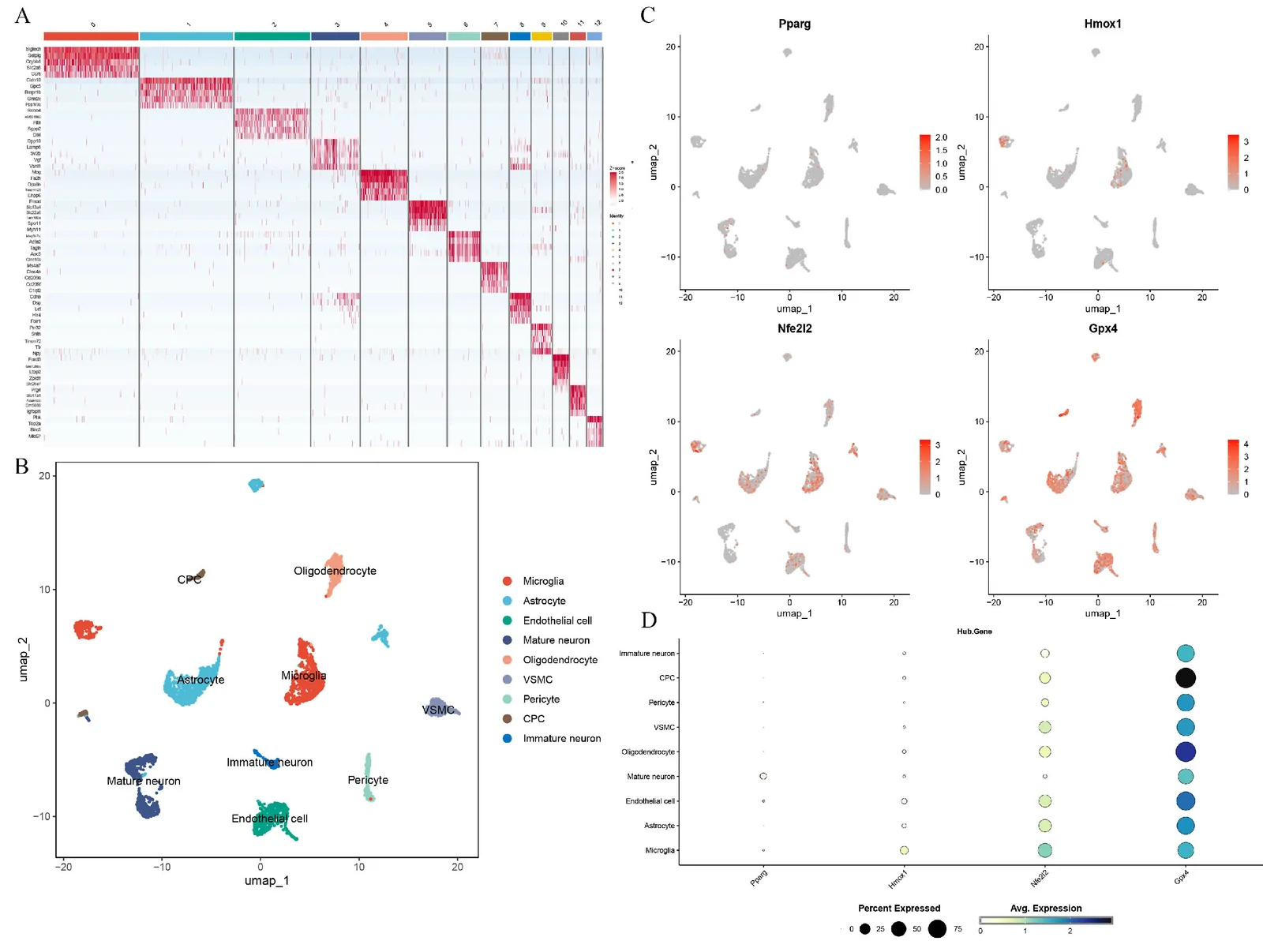
scRNA-seq analysis of hippocampal cell populations and key gene expression (GSE201644). (**A**) Marker gene expression heatmap. (**B**) UMAP plot of 9 major hippocampal cell types. (**C**) Feature plots of hub genes (*Pparg*, *Hmox1*, *Nfe2l2*, *Gpx4*). (**D**) Bubble plot showing cell type-specific hub gene expression patterns.

**Figure 3:**
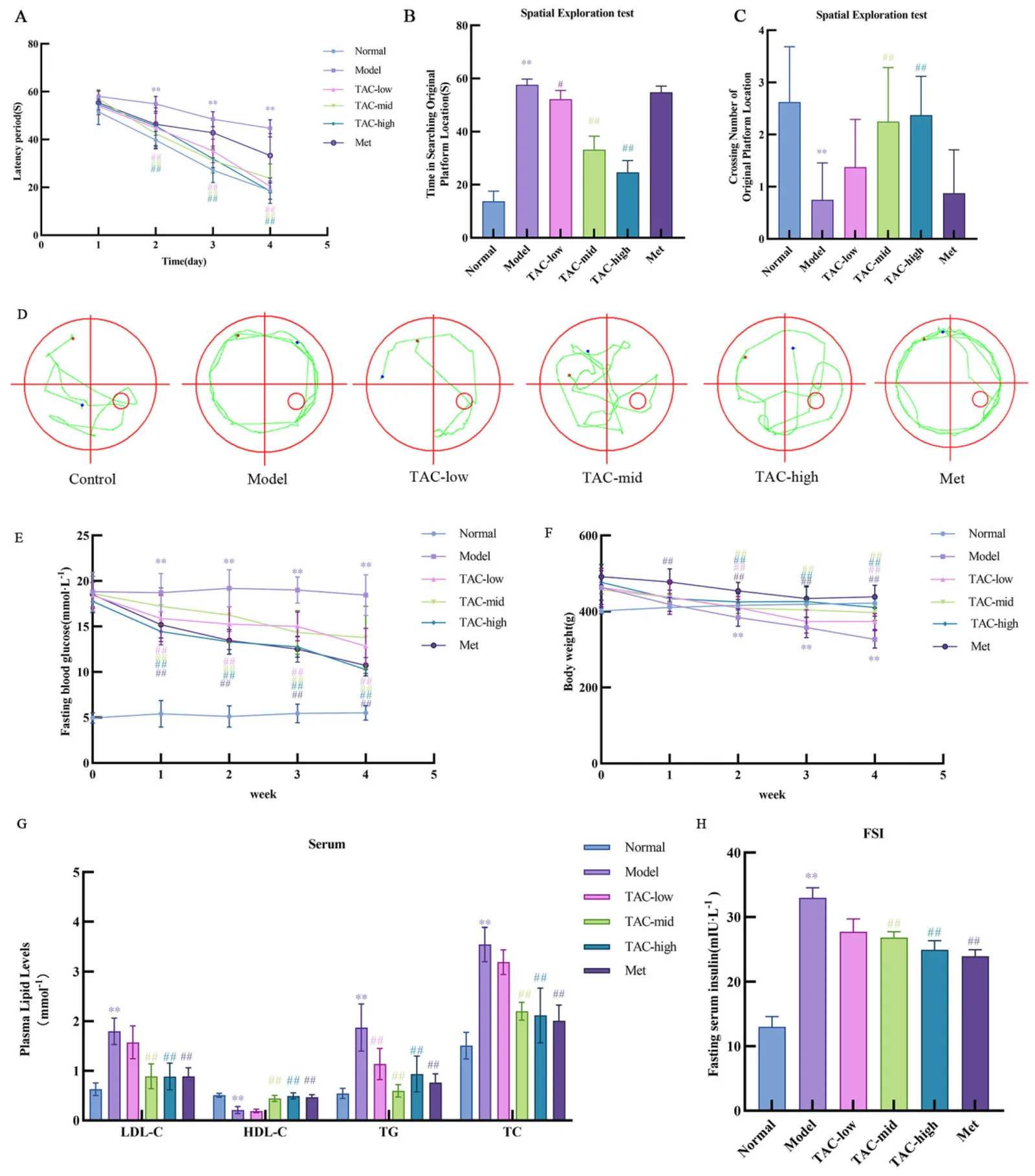
TAC improves cognitive deficits, hyperglycemia, and weight loss in diabetic rats (TAC-L: 7 mg·kg^−1^, TAC-M: 11 mg·kg^−1^, TAC-H: 14 mg·kg^−1^). (**A**) Escape latency (MWM training). (**B**–**D**) Spatial probe test: target quadrant time, platform crossings, and representative paths. (**E**,**F**) Fasting blood glucose and body weight changes. (**G**) Serum lipid profiles (LDL-C, HDL-C, TG, TC). (**H**) Fasting serum insulin (FSI) levels. Mean ± SD. n = 8. Statistical analysis: one-way or two-way ANOVA with LSD method. ** *p* < 0.01 vs. Normal group, # *p* < 0.05 vs. Model Group, ## *p* < 0.01 vs. Model Group.

**Figure 4:**
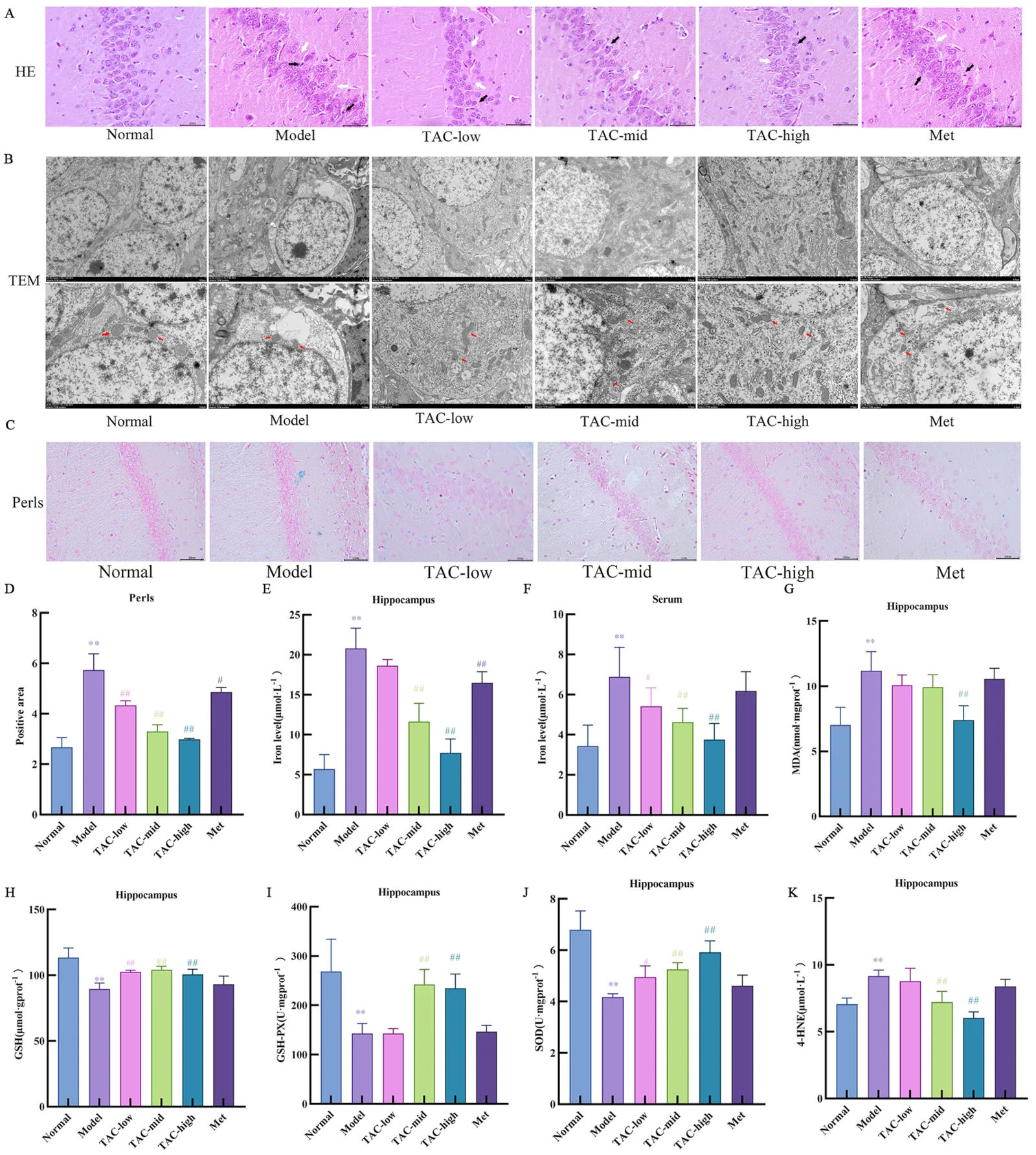
TAC alleviates iron overload and oxidative stress in the hippocampus of diabetic rats (TAC-L: 7 mg·kg^−1^, TAC-M: 11 mg·kg^−1^, TAC-H: 14 mg·kg^−1^). (**A**) Hippocampal HE staining (scale bar: 200 μm). Black arrow: nuclear pyknosis. White arrow: neuronal degeneration and losses. (**B**) Hippocampal neuron ultrastructure (scale bar: 2 μm and 5 μm, n = 3). Red arrow: mitochondria. (**C**) Perls staining for hippocampal iron deposition (scale bar: 200 μm, n = 3). (**D**) Quantification of Perls-positive area (n = 3). (**E**) Hippocampal iron level (n = 8). (**F**) Serum iron level. (**G**–**K**) Hippocampal MDA (**G**), GSH (**H**), GSH-Px (**I**), SOD (**J**), and 4-HNE (**K**) levels (n = 8). Mean ± SD. Statistical analysis: one-way ANOVA with LSD method. ** *p* < 0.01 vs. Normal group; # *p* < 0.05, ## *p* < 0.01 vs. Model group.

**Figure 5:**
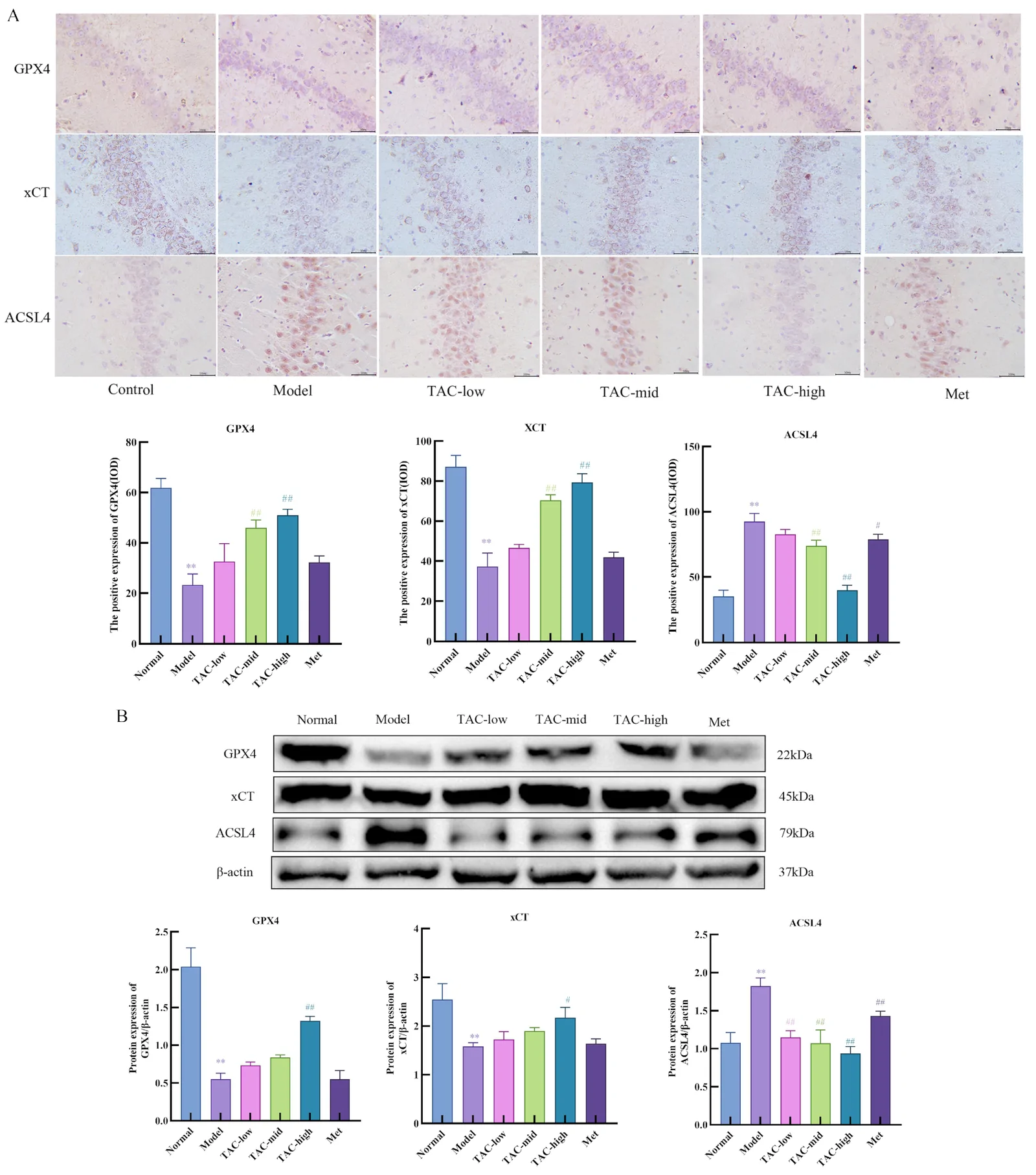
TAC modulates ferroptosis-related protein expression in the hippocampus of diabetic rats (TAC-L: 7 mg·kg^−1^, TAC-M: 11 mg·kg^−1^, TAC-H: 14 mg·kg^−1^). (**A**) IHC staining and quantification of GPX4, xCT, and ACSL4 in hippocampus (scale bar: 200 μm). (**B**) Western blot and quantification of GPX4, xCT, and ACSL4 (β-actin as loading control). Mean ± SD (n = 3). Statistical analysis: one-way ANOVA with LSD method. ** *p* < 0.01 vs. Normal; # *p* < 0.05, ## *p* < 0.01 vs. Model.

**Figure 6:**
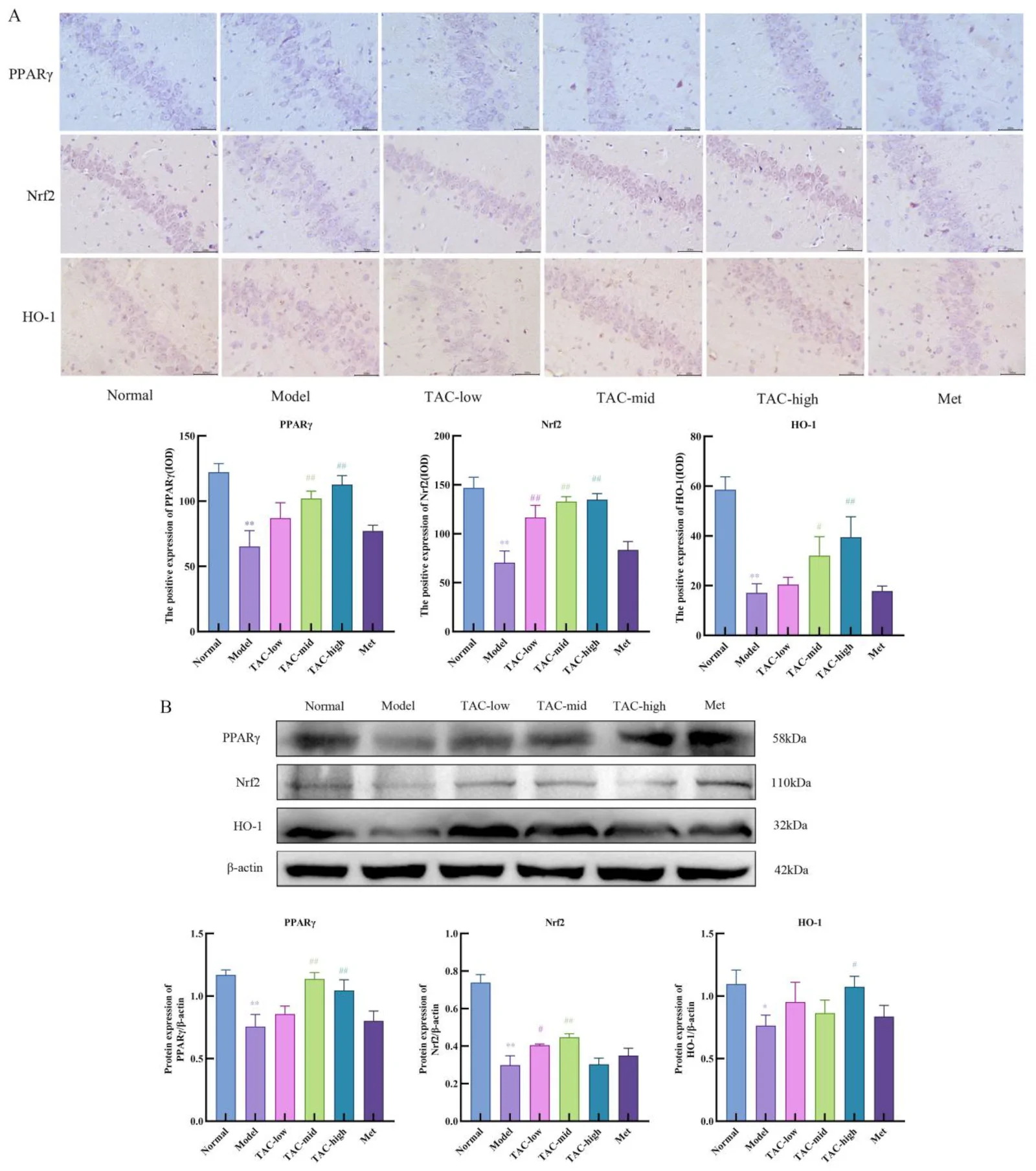
TAC upregulates the PPARγ/Nrf2/HO-1 pathway in diabetic rat hippocampus (TAC-L: 7 mg·kg^−1^, TAC-M: 11 mg·kg^−1^, TAC-H: 14 mg·kg^−1^). (**A**) IHC staining and quantification of PPARγ, Nrf2, HO-1 in hippocampal tissue (scale bar: 200 μm). (**B**) Western blot and quantification of PPARγ, Nrf2, HO-1 (β-actin as loading control). Mean ± SD (n = 3). Statistical analysis: one-way ANOVA with LSD method. * *p* < 0.05, ** *p* < 0.01 vs. Normal; # *p* < 0.05, ## *p* < 0.01 vs. Model.

**Figure 7:**
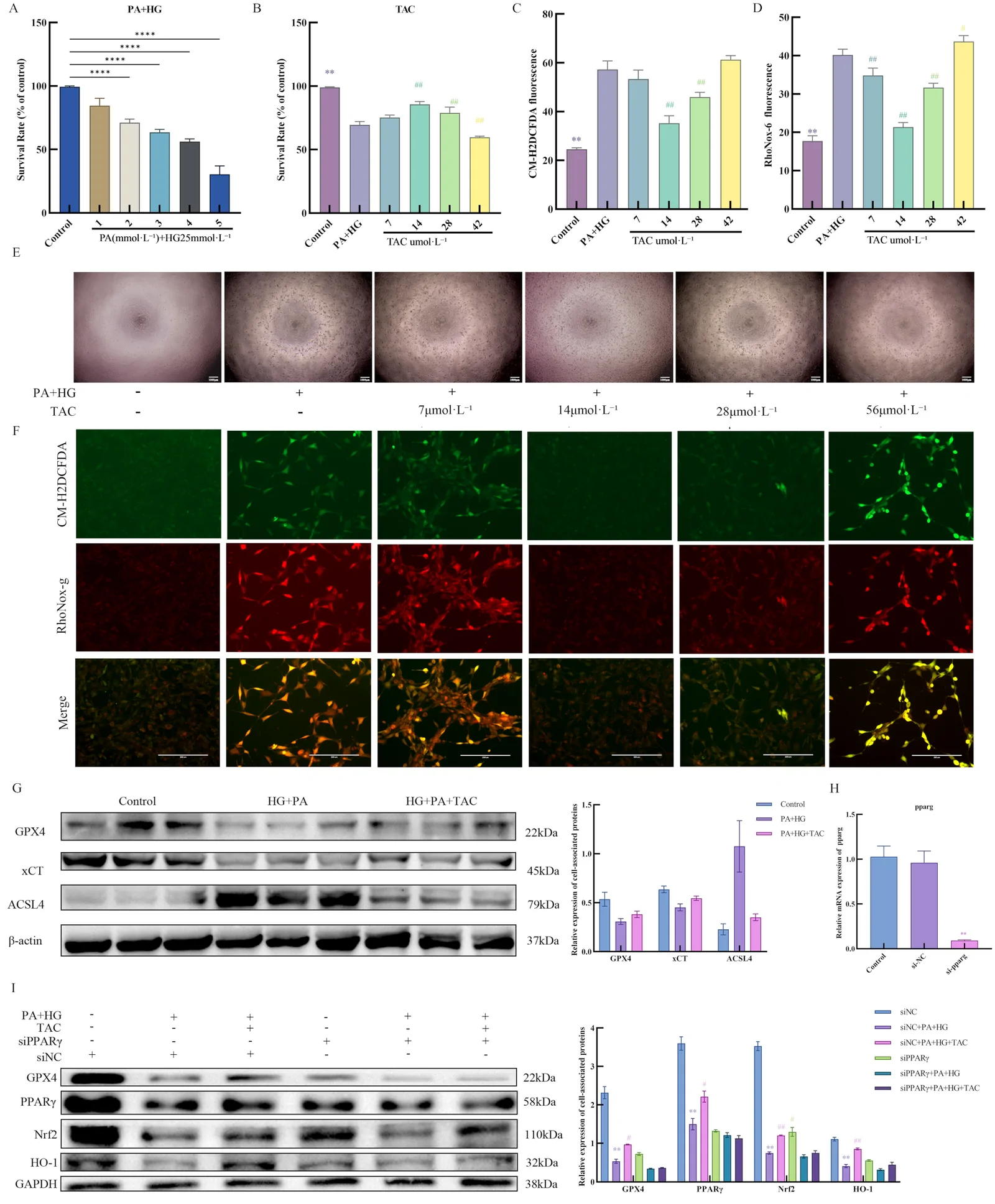
Optimization of PA + HG modeling and TAC-containing serum concentrations in vitro. (**A**) HT22 cell viability under different PA + HG concentrations (1–5 mmol·L^−1^ PA + 25 mmol·L^−1^ HG). (**B**,**E**) Viability of PA + HG-treated HT22 cells intervened with 7–42 μmol·L^−1^ TAC-containing serum(scale bar: 1000 μm). (**C**,**D**) Quantitative fluorescence analysis of ROS (CM-H2DCFDA) and mitochondrial superoxide anion (RhoNox-6). (**F**) Representative CM-H2DCFDA, RhoNox-6, and merged fluorescence images (scale bar: 200 μm). (**G**) Western blot and quantification of GPX4, xCT, ACSL4 (β-actin as loading control). (**H**) q-PCR analysis of PPARγ mRNA expression after siRNA transfection. (**I**) Western blot and quantification of GPX4, PPARγ, Nrf2, HO-1 (GAPDH as loading control). Mean ± SD (n = 3). Statistical analysis: one-way ANOVA with LSD method. ** *p* < 0.01, **** *p* < 0.0001 vs. Control group; # *p* < 0.05, ## *p* < 0.01 vs. siNC + PA + HG group.

## Data Availability

All data generated or analyzed during this study are included in this published article. The data that support the findings of this study are openly available in the Gene Expression Omnibus at https://www.ncbi.nlm.nih.gov/geo/, accessed on 2 May 2026, reference number GSE201644.

## Supplementary Materials

The following supporting information can be downloaded at https://www.mdpi.com/article/10.3390/ph19101506/s1. Table S1: TAC active ingredient–potential target docking results.
