# Supplementary material for: Protective Effects of Total Alkaloids of Rhizoma Corydalis Against Neuronal Ferroptosis Through a PPARγ-Dependent Mechanism in Diabetes-Associated Cognitive Impairment
Source: Pharmaceuticals (Basel). 2026 Sep 22;19(10):1506. doi: 10.3390/ph19101506 (PMC13708249; doi:10.3390/ph19101506)
Supplement: Supplementary file 1 [file pharmaceuticals-19-01506-s001.zip › pharmaceuticals-4449296-supplementary.pdf]

Table S1 TAC active ingredient – potential target docking results

Affinity values（KJ • mol^{-1}）	TAC active ingredient		
	**Isocorypalmine**	**Leonticine**	**(s)-Scoulerine**
ESR1	−6.863	−8.341	−6.815
PPARγ	-8.982	-7.962	-8.899
FOS	−5.648	−5.291	−5.735
CREB1	−5.871	−5.743	−5.971
BDNF	−6.459	−5.804	−5.144
